# Choice of Leisure Activities by Adolescents and Adults With Internet Gaming Disorder: Development and Feasibility Study of a Virtual Reality Program

**DOI:** 10.2196/18473

**Published:** 2020-12-11

**Authors:** Narae Lee, Jae-Jin Kim, Yu-Bin Shin, Hyojung Eom, Min-Kyeong Kim, Sunghyon Kyeong, Young Hoon Jung, Sarang Min, Joon Hee Kwon, Eunjoo Kim

**Affiliations:** 1 Department of Psychiatry & Institute of Behavioral Science in Medicine Yonsei University College of Medicine Seoul Republic of Korea; 2 Department of Psychiatry Yonsei University Gangnam Severance Hospital Seoul Republic of Korea; 3 Brain Korea 21 PLUS Project for Medical Science Yonsei University Seoul Republic of Korea

**Keywords:** virtual reality, VR, internet game disorder, IGD, cognitive behavioral therapy, CBT, motivation

## Abstract

**Background:**

Excessive internet game use frequently leads to various physical, psychological, and social problems, and internet gaming disorder (IGD) has become a serious public health issue worldwide. Recently, virtual reality (VR) therapy has emerged as a promising method to increase psychological treatment motivation and accessibility. However, few studies have examined the potential of VR technology for the management of IGD, and VR content tailored to IGD characteristics remains scarce.

**Objective:**

This preliminary study aimed to examine the potential of a VR-based program that was designed to help users identify their leisure time use patterns, especially those related to gaming, and to modify their gaming overuse by alternative activities provided in the VR content. Moreover, to investigate whether users’ VR activities reflect various clinical variables of IGD in youth, we examined the relationships among the leisure time activity selection pattern, built-in response, and speech data obtained from the VR program, as well as symptom severity of internet gaming, psychiatric comorbidities, and motivation of participants reported through relevant questionnaire data.

**Methods:**

Three types of VR content (understanding my daily activities at home, finding an alternative activity to internet gaming at home, expressing contradictory opinions toward a friend’s gaming beliefs) were developed by simulating the daily situations in which patients with IGD can select alternative free-time leisure activities. We examined internet addiction, mental health problems, and motivation for 23 IGD and 29 control participants. Behavioral and self-rated responses from VR, such as alternative activity selection data and speech patterns (speech time, speech satisfaction, and speech accordance), and results from various questionnaires were compared between groups. The correlations between IGD behaviors in VR and real-life behaviors assessed by questionnaire measures were analyzed.

**Results:**

Significant correlations were found between internet gaming behavior and user activity data, such as speech and activity selection pattern, in our VR program. Our results showed that the IGD group had fewer leisure activities and preferred game or digital activities to other types of activities compared to controls, even in VR. There was a positive relationship between the viability of alternative leisure activities the participants selected in VR and the amount of perceived satisfaction from that activity (r=.748, *P*<.001). Speech accordance in the IGD group was lower than in the control group and was correlated negatively with Internet Addiction Test and Internet Addiction Test–gaming scores (r=.300, *P*=.03) but positively with users’ motivation (r=.312, *P*=.02).

**Conclusions:**

The results from our VR program can provide information about daily activity patterns of youths with IGD and the relationship between user VR activities and IGD symptoms, which can be useful in applying VR technology to IGD management.

## Introduction

Internet gaming is the most common leisure activity among adolescents in East Asian countries, including South Korea [1,2] and China [3]. While some studies have proposed positive effects of internet gaming [4,5], excessive internet game use frequently leads to various physical, psychological, and social problems [6-8]. The concept of internet gaming disorder (IGD), deﬁned as the persistent and recurrent use of the internet to engage in games despite negative consequences, has been controversial. Diagnostic criteria or guidelines for IGD have been proposed by several investigators, but there has been continued debate around the conceptualization of IGD. Therefore, studies of IGD-targeted treatment mechanisms are needed to help with the classification of behavioral addiction and the effective management of individuals with IGD [9-11].

Regarding IGD treatment, theories based on cognitive behavioral therapy (CBT) and motivational enhancement therapy (MET) have been promising approaches to explain addictive behaviors [12], and they have been commonly used as effective treatment for IGD [12-14]. The CBT model claims that the identification and modification of maladaptive cognition on internet gaming are crucial factors for treatment [14]. Maladaptive cognitions such as excessive preoccupation with internet gaming, impaired cognitive control, and cognitive inflexibility contribute to compulsive internet gaming activity [13,15,16].

Additionally, the motivation-focused model of addiction has been employed for the management of IGD, as motivational drives linked to reward-seeking also contribute to problematic gaming behavior [15,17]. Individuals with IGD are reported to have enhanced reward sensitivity and decreased loss sensitivity, leading to stronger motivation to play compared to people without IGD [15,16,18].

Taken together, CBT and MET models of addiction suggest that treatment for IGD requires assessing the individual’s cognitive and behavioral patterns and motivation before a specific therapeutic plan is implemented. Therefore, the management of the selection process for gaming activities is important for finding the triggering factors that motivate and maintain people’s excessive gaming behaviors [6,19]. Since people with IGD often do not have insight on their excessive preoccupation with gaming, it is necessary for patients with IGD to identify the pattern and underlying motivation of their leisure activities to manage their problematic gaming effectively. For example, identification of the times and places that elicit people’s craving for gaming in their free time and a technique to control this craving and the motivational components of internet gaming is necessary for IGD management [20]. For this purpose, therapists also need to observe and intervene in the behavior of individuals with IGD in response to exposure cues for leisure activities in situations that increase the patients’ risk of gaming. However, it is difficult to reproduce the actual reality of the gaming cue exposure through the process of traditional treatment. Besides, in a real-world situation, it is also difficult to follow up and collect patients’ behavioral data in real time.

Alternatively, CBT using virtual reality (VR) technology is a promising method to increase motivation for and accessibility of mental health treatments, especially among youth who are generally positive to the appeal of modern digital technology [12-14,21-23]. Moreover, VR features are immersive and interactive, simulating reality without space and time limits [24]. Gestures and voice inputs are effective means of interacting with avatars in virtual space while also maintaining full potential for flow and immersion [24,25]. The VR program also automatically records users’ activity and verbal data, allowing for real-time data analysis. Despite these advantages of VR-CBT, few studies have examined the effects of VR for IGD management, and VR content tailored to IGD characteristics is scarce.

Regarding the embodiment of IGD management techniques, we mainly used the self-speech technique. The definition of “self-speech” in our program was the verbalization of one’s opinions in their own words in response to the questions asked by avatars. Self-speech is reported to help to self-regulate cognition and behavior in addition to positively influencing self-control [26,27]. It also helps people accomplish self-distancing and objectification, enabling individuals with IGD to reappraise their cognition on gaming guided by objective viewpoints in the VR program [28]. As computer technologies related to voice recognition and processing have developed, speech has also become a viable interaction modality in VR environments.

Given this background, the objectives of this study were to examine the potential of a VR-based program that was designed to help users identify their leisure time use patterns, especially those related to gaming, and to modify their gaming overuse by alternative activities provided in the VR content. More specifically, to investigate whether behavioral responses to our VR program were associated with various IGD clinical variables, we examined the relationships among leisure time activity selection pattern, built-in response and speech data (speech time, speech satisfaction, and speech accordance) obtained from our VR program, and IGD symptom severity, psychiatric comorbidities, and motivation of participants reported through relevant questionnaire data.

## Methods

### Sample Size (Power)

Power analysis for an independent *t* test was conducted in the G*Power program to determine the necessary sample size, with an alpha of .05, power of 0.80, large effect size (f =0.8), and 2 tails [29]. The results from our sample size calculation suggested that the desired sample size was estimated to be 26 in each group, totaling 52 subjects, to detect the group difference in our dependent measures.

### Recruitment

Participants were recruited from online community and social network sites in South Korea and through flyers. Since men are known to have a higher prevalence of IGD than women, and to avoid gender-specific confounding factors related to the pattern of gaming craving and motivations affecting the results, only male participants were recruited [30]. Participants were 52 men aged 11-25 years, and 44 (44/52, 85%) were either high school or university students, aged between 16 and 25 years. Age was matched between the 2 groups during the assignment procedure, but other variables in our study were the result of simple randomization. All participants were evaluated for internet use patterns and interviewed by a psychiatrist for IGD diagnosis according to the Diagnostic and Statistical Manual of Mental Disorders, Fifth Edition (DSM-5) [31]. In the control group, no one had ever been diagnosed with IGD or other DSM-5 psychiatric disorders. Exclusion criteria for all participants were current use of psychotropic medication and history of substance use disorder, serious neurological or medical disorder, bipolar I disorder, and psychotic disorder according to the Mini-International Neuropsychiatric Interview for Children and Adolescents (MINI-KID 6.0) [32] for participants younger than 18 years and the MINI 5.0 for adult participants [33]. However, 3 participants with psychiatric comorbidities were included (1 attention deficit hyperactivity disorder [ADHD], 1 depressive disorder, 1 adjustment disorder) in the IGD group.

### Measures

IQ was assessed using the short form of the Wechsler Intelligence Scale for Children—Third Edition [34] and Wechsler Adult Intelligence Scale—Revised [35,36]. The Internet Addiction Test (IAT) [8], one of the most utilized diagnostic instruments for internet addiction, was used to assess IGD symptom severity (Cronbach α=.90). All participants completed a modified version of the IAT that replaced the term “Internet” with terms such as “Internet games” to assess specific subtypes of internet addiction [37-39]. As IGD is frequently comorbid with other psychiatric disorders [40], participants completed the short version of Conners-Wells’ Adolescent Self-Report Scale (CASS-S) for adolescents (Cronbach α=.88) [41] and Conner’s Adult ADHD Rating Scale (Cronbach α=.87) [42] for adult participants to measure ADHD symptoms [42,43], Center for Epidemiological Studies Depression Scale (CES-D) for depression (Cronbach α=.91) [44,45], and Beck Anxiety Inventory (BAI) for anxiety (Cronbach α=.92) [46]. The Free Time Motivation Scale for Adolescents (FTMS-A; Cronbach α=.75) was also administered to measure 5 subscales representing various types of motivation to engage in certain types of leisure time activities [47].

The presence questionnaire (PQ; Cronbach α=.84) [48,49], Simulator Sickness Questionnaire (SSQ) [50], and Client Satisfaction Questionnaire (CSQ) [51] were used to measure the users’ experience with our system. PQ is a measure of users’ presence in virtual reality, which is a psychological state of “being there” and an important element of VR experience. Presence can enhance users’ active engagement in content, involving their senses and capturing their attention. SSQ assesses simulator sickness resulting from the discrepancy between simulated visual motion and the sense of movement coming from the vestibular system. Simulator sickness is negatively correlated with users’ enjoyment of VR programs. Finally, CSQ was used to rate the users’ general satisfaction with our VR training session.

### Procedures

A between-subjects user study was conducted for about 1 hour. Participants were asked to complete written questionnaires about their demographic background and internet usage patterns, including the average hours they spent online gaming, frequency of internet usage, and name of a game they mainly play. Participants were adjusted to the virtual environment during the training session and then were asked to play 3 types of VR content, described in the following sections. The content was counterbalanced to prevent systemic order effect. Upon completing the program, participants were debriefed about their VR experience, using the postquestionnaire form.

### Virtual Reality

#### System Design

The VR system was a desktop computer running Microsoft Windows 10 containing an NVIDIA GeForce GTX 970 graphics card and an Oculus Rift head-mounted display with tracker (Oculus VR, LLC, Irvine, CA; HD resolution of 1080 × 1200 per eye with 51.6 diagonal field of view, a 3-DOF tracker for head rotation, and built-in headphones). Mounted on the headset, the Leap Motion Controller (Leap Motion Inc, San Francisco, CA), a new device suitable for hand gesture–controlled user interfaces, was used for interactions with executable objects and avatars during the VR experience (Multimedia Appendix 1). The microphone built into the VR headset gathered verbal data from users’ self-speech in real time. Three-dimensional virtual environments included a virtual house and appliances using Autodesk 3D max. User instruction was provided verbally. The virtual environment was integrated with Unity software. User data such as type of selected activities and selection time, head movement, hand gesture, speech content, and speaking time were recorded and stored in the main server computer. Menu navigation on screen was performed by virtual hands positioned to match the user’s hands. This system allowed the therapist to track patient performance and analyze behavioral information.

#### Content Design

Our VR training system emphasized the development of motivation for change based on the implementation of CBT and MET principles by: (1) identifying situations, thoughts, and behavior associated with internet game use by providing situations that increase the patients’ risk of gaming and dysfunctional beliefs that support the maintenance of behavioral addiction issues; (2) conducting cognitive restructuring with these dysfunctional beliefs by describing the pros and cons of his or her online activity, expressing contradictory opinions against irrational beliefs about games; and (3) raising one’s motivation by devising a self-determined choice of activities to modify one’s maladaptive leisure time use pattern.

**Figure 1:**
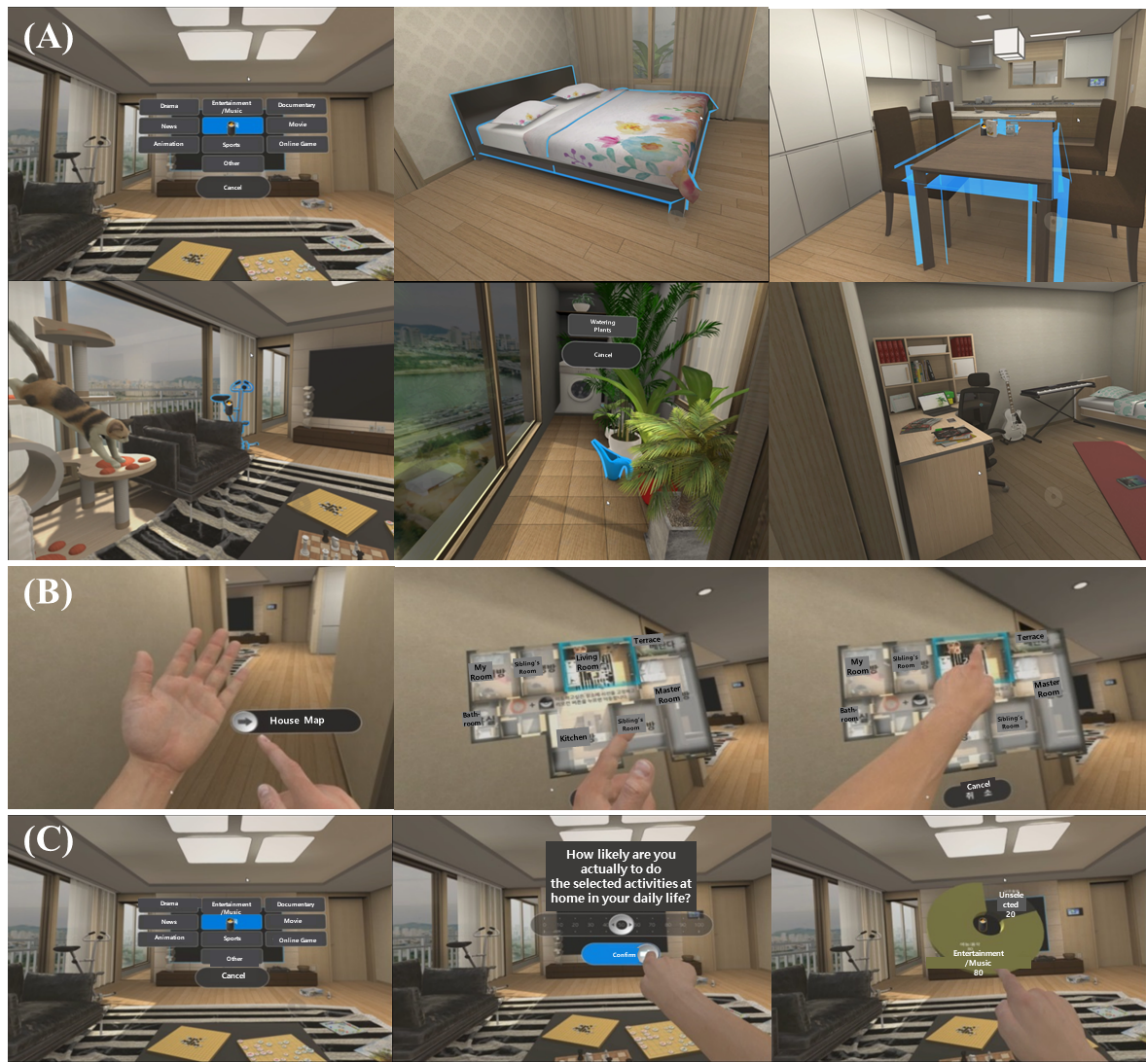
Screenshots of apartment setting (A), gestural input (B), and questionnaire (VAS) and visual feedback for selected activities (C) in VR.

Content 1 involved “understanding my daily leisure activities at home” and was designed to understand the pattern of participants’ choice of leisure activities in daily life. It provided real-life situations that increase the patients’ risk of internet gaming and their dysfunctional behavioral patterns that support the maintenance of gaming issues. Figure 1A shows a blueprint menu of an indoor apartment showing the whole space for finding leisure activities. It consisted of a porch, participant’s room, small room, living room, kitchen, and restroom. At the start of each level, the entrance of the apartment was displayed from the first-person perspective. When users selected a room in the House Map menu with their hands, the scene automatically started at the entrance of the chosen room. The program then suggested users explore and select a set of leisure activities by choosing virtual objects placed in each room (Figure 1B). After selecting each activity, 2 questions were presented on the head-mounted display screen. Users determined the proportion of the chosen activity during daily life measured using a visual analog scale (VAS) ranging from 0% to 100%. Missions A and B consisted of the same virtual environment, but Mission A aimed to discover leisure activities during the daytime, while Mission B targeted activities during the nighttime. Multimedia Appendix 2 explains the list of leisure activities users can select in each room.

Content 2 involved “finding an alternative activity to internet gaming at home” and was designed to encourage users to discover alternative activities the user can substitute for game playing. This is analogous to the alternative thought recording, which is one of the most commonly used CBT techniques to modify an individual’s irrational cognition. We tried to figure out the behavioral differences between the IGD group and control group through the alternative activity selection pattern in VR and self-reported viability. The virtual environment of Content 2 was identical to that of Content 1. The program asked users to find leisure activities commonly found at home to replace internet gaming. Once an activity was selected, the self-assessment for perceived viability (“How likely are you actually to do the selected activities at home in your daily life?”) and perceived satisfaction (“How satisfied do you think you will be when you participate in the selected activity?”) of the selected activity were measured using a VAS (Multimedia Appendix 3). Additionally, an Add Activity menu was provided to add custom activities that are not found in the apartment to maximize user autonomy. After choosing all leisure activities, visualized training feedback results that were analyzed based on the participants’ own activities in VR were displayed as a pie diagram (Figure 1C). The program also automatically recorded activity type and corresponding response, allowing for real-time data analysis. The activities on a computer, mobile phone, and console game were included in the digital activity category for analysis.

Content 3 involved “expressing contradictory opinions toward friend’s gaming beliefs” and was designed for users to reappraise their cognition regarding games, rebutting common motivations for playing games. In Mission A, we collected participants’ self-speech data in which they express contradictory opinions against male avatar’s beliefs about games and recommend alternative activities they chose in Content 2 to the avatar friends in VR. Then, users were asked to express contradictory opinions toward the avatar peers’ beliefs regarding games through self-speech about 6 topics that are based on gaming motivation theory of socialization, achievement, and dissociation (see the detailed scripts of these topics in Multimedia Appendix 4). Also, the users were given a VR content wherein an avatar peer-pressured the user to play a game together to relieve stress from exams or to win a battle against their opponents. The users were instructed to refuse the avatar’s invitations to play the game by stating the reason or to provide alternative activities to gaming; whether the users could refuse their peers’ proposals to play the game was observed. Through this training, users could also review their gaming motivations and ensure that self-assertiveness is achieved properly.

In Mission B, the users’ own selection of alternative activities from Content 1 and Content 2 were displayed as a mind map on the TV screen, helping users to make a 1-minute speech about the strengths of alternative activities on physical, social, and psychological aspects in comparison to gaming and internet use. All the speech content was automatically recorded when users pressed a speech button during the VR program. After each speech session, participants were required to rate their speech using a VAS in terms of 3 elements: speech time (duration of speech measured in milliseconds), speech satisfaction (self-assessment of satisfaction of one’s speech), and speech accordance (concordance of self-speech contents they spoke and users’ actual thoughts; “How much does your opinion in reality match what you just said?”).

### Statistical Analysis

The main dependent variables in each group were normally distributed, as assessed by a Shapiro-Wilk test (*P*>.05). All demographic and clinical symptom measures were compared between the IGD and control groups using independent *t* tests (2-tailed). Bivariate correlation analysis (Pearson r) was conducted to examine the association between behavioral and speech results on VR tasks and IGD symptom severity assessed by IAT and IAT-gaming and various clinical questionnaire measures, such as CASS-S, CES-D, BAI, and FTMS-A. All analyses were conducted using SPSS 20.0 (SPSS Inc, Chicago, IL). In all cases, *P*<.05 (2-tailed) was considered statistically significant.

### Ethics

The current study was approved by the Institutional Review Board of Yonsei University College of Medicine, Gangnam Severance Hospital, and written informed consent was obtained from all participants after they had received a detailed explanation of the study. In case of adolescents, written informed consent of their parents was also obtained.

## Results

### Comparison of Clinical Symptom Measures Between IGD and Control Groups

Table 1 shows the result of the 2-tailed independent *t* tests between the IGD and control groups in demographic and clinical variables. There were no differences in age and IQ between groups. As expected, the IGD group had higher IAT and IAT-gaming scores. A significantly higher CASS-S score was also reported for the IGD group, suggesting that the IGD group showed higher ADHD symptoms compared to controls. Moreover, IAT and BAI (r_52_=.314, *P*=.02), IAT and CES-D (r_52_=–.274, *P*=.049), IAT-gaming and BAI (r_52_=.339, *P*=.01), and IAT-gaming and CES-D (r_52_=–.260, *P*=.06) were significantly correlated, indicating that the severity of anxiety and depression symptoms was significantly associated with IGD severity.

**Table 1:** Demographic and clinical characteristics of participants.

Characteristics	IGD^a^ group, (n=23), mean (SD)	Control group, (n=29), mean (SD)	*t* test^b^	*P* value
Age (years)	18.91 (3.87)	19.75 (3.87)	0.788	.44
IQ	113.6 (14.37)	117.6 (13.06)	0.038	.32
IAT^c^	64.87 (15.53)	37.68 (11.58)	−6.998	.00
IAT-gaming	64.23 (15.09)	35.08 (12.76)	−8.940	.00
CES-D^d^	14.83 (9.01)	10.83 (8.07)	−1.860	.07
BAI^e^	7.09 (7.39)	3.76 (4.05)	−2.068	.44
ADHD^f^ score	26.61 (14.73)	16.17 (9.03)	−3.146	.003
PQ^g^	122.87 (26.67)	133.10 (17.84)	1.654	.10
SSQ^h^	24.35 (7.64)	25.28 (10.26)	0.361	.72
CSQ^i^	11.65 (2.85)	13.31 (2.46)	2.246	.29

^a^IGD: internet gaming disorder.

^b^The degrees of freedom for all *t* tests were 51.

^c^IAT: Internet Addiction Test.

^d^CES-D: Center for Epidemiological Studies Depression Scale.

^e^BAI: Beck Anxiety Inventory.

^f^ADHD: Attention Deficit Hyperactivity Disorder.

^g^PQ: Presence Questionnaire.

^h^SSQ: Simulator Sickness Questionnaire.

^i^CSQ: Client Satisfaction Questionnaire.

### Leisure Activity in VR Content and Symptoms of IGD

**Figure 2:**
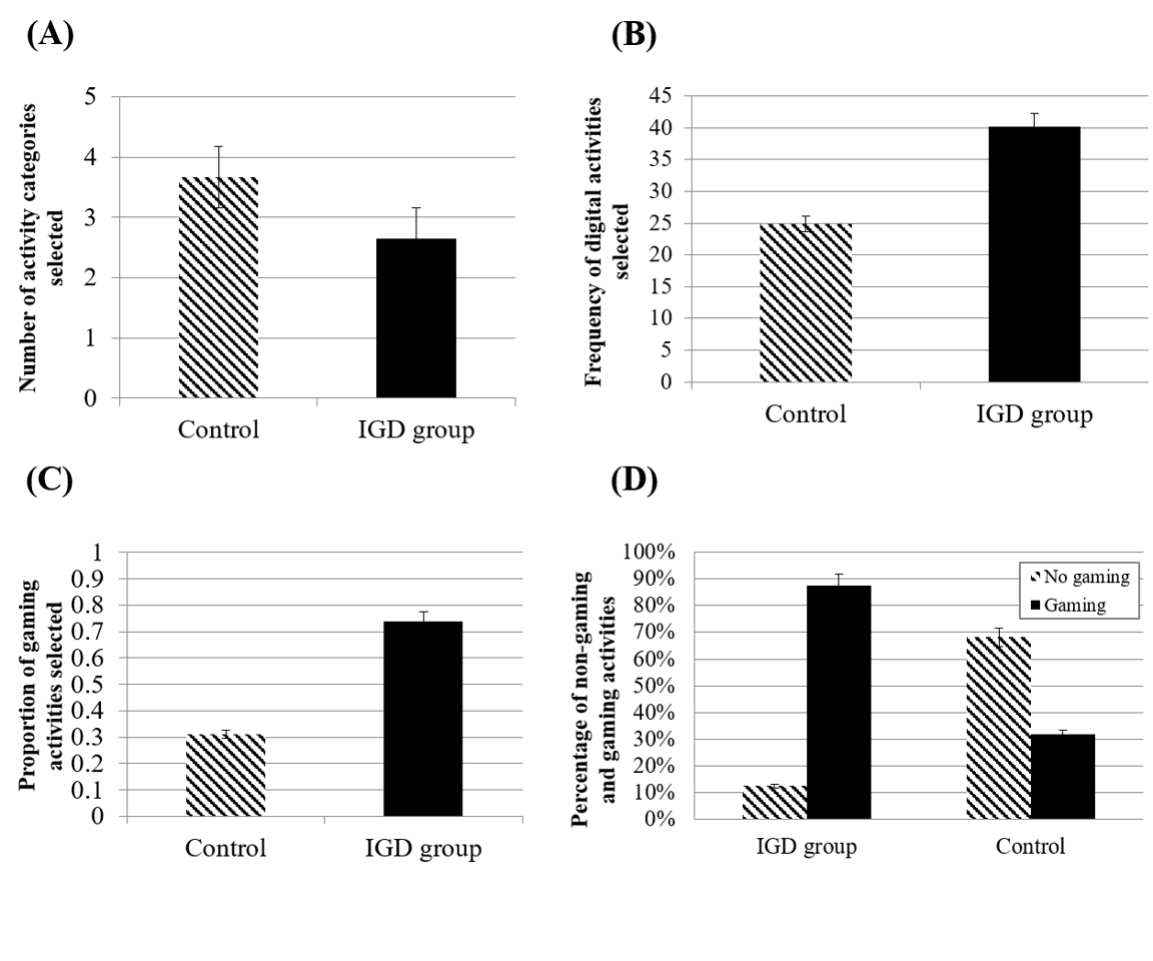
Frequency of digital and game activities in the virtual reality environment in Content 1: (A) number of selected activity categories in level 1, (B) frequency of the selection of digital and gaming activities, (C) proportion of the selection of gaming activities, (D) percentage of nongaming and gaming activities in each group. IGD: internet gaming disorder.

In Content 1, we tried to analyze the number and patterns of daily leisure activities in VR in the IGD and control groups. Our results showed that the IGD group had fewer activity domains than controls (t_51_=3.529, *P*<.001; Figure 2A). Interestingly, even in the VR content, there were significant differences in the percentage of digital or game activities (ratio of game or digital leisure activities to total selected activities) between the IGD group and controls (t_51_=−2.79, *P*=.007; Figures 2B and 2C). The IGD group preferred game or digital activities to other types of leisure activities more than controls did, and chosen activities were mostly in the mobile and computer category rather than being varied (Figure 2D).

**Figure 3:**
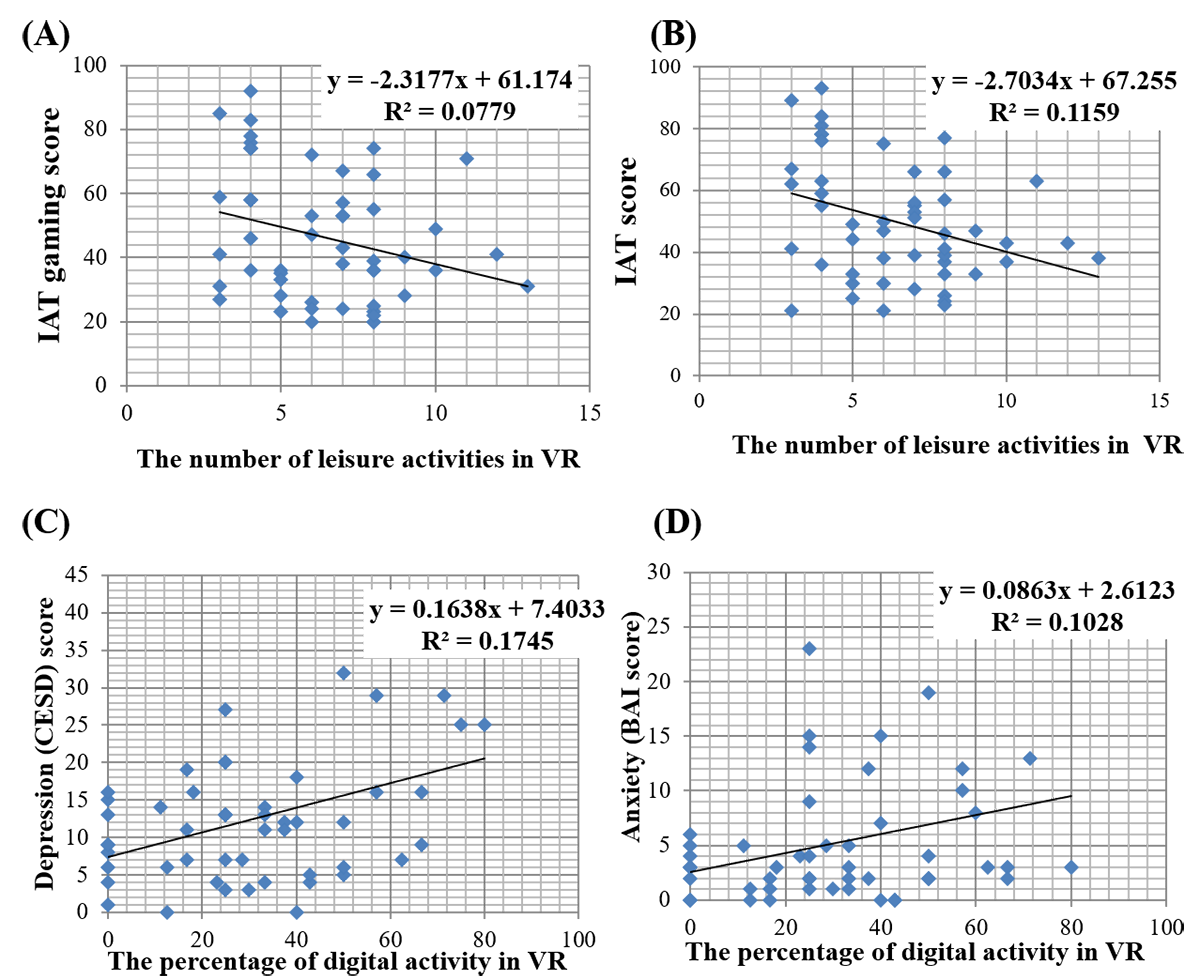
Relationships between the Internet Addiction Test (IAT) gaming score and (A) number of leisure activities in the virtual reality (VR) environment and (B) IAT score as well as relationships between the percentage of digital activity in the VR environment and (C) depression and (D) anxiety. BAI: Beck Anxiety Inventory; CES-D: Center for Epidemiological Studies Depression Scale.

In Content 2, correlation analysis showed that the number of alternative activities chosen by each participant was negatively correlated with both the IAT (r_52_=−.341, *P*=.01) and IAT-gaming (r_52_=−.280, *P*=.04) scores (Figure 3A). Additionally, the percentage of digital activities in VR was positively related to depression (r_52_=.416, *P*=.002) and anxiety (r_52_=.321, *P*=.02; Figure 3B), indicating that selected activity in VR can closely relate to psychiatric symptoms. Lastly, there was a positive relationship between the viability of alternative leisure activity participants selected in the VR environment and the amount of perceived satisfaction from that activity (r_52_=0.748, *P*<.001).

### Speech Patterns About the Choice of Leisure Activities in VR

**Figure 4:**
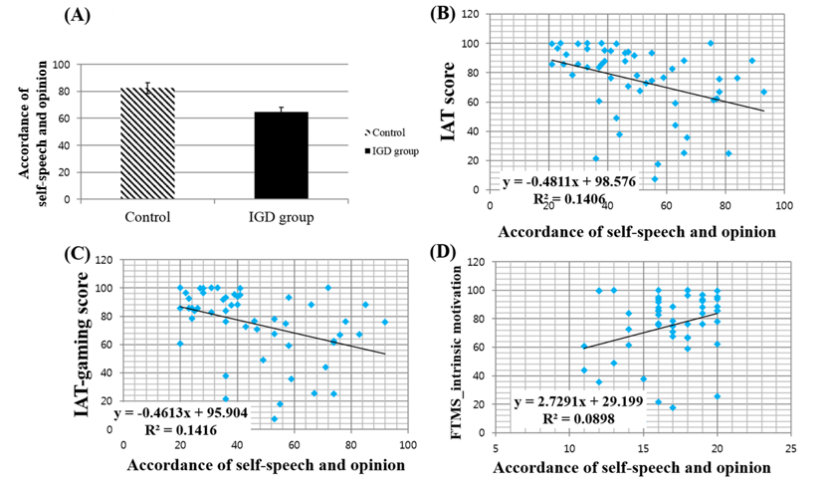
(A) Comparison of the accordance of self-speech contents and opinion between the control and internet gaming disorder (IGD) groups and correlations between accordance of self-speech and opinion and (B) Internet Addiction Test (IAT) score, (C) IAT-gaming score, and (D) Free Time Motivation Scale (FTMS)-intrinsic motivation.

In Content 3, the independent *t* tests showed that users’ speech time and speech satisfaction were not significantly different between the IGD and control groups, but speech accordance in the IGD group was lower than in the control group (t_51_=2.762, *P*=.008; Figure 4A). Additionally, speech accordance was negatively correlated with IAT (r_52_=–.375, *P*=.006; Figure 4B) and IAT-gaming scores (r_52_=–.376, *P*=.006; Figure 4C), but positively correlated with motivation (r_52_=.300, *P*=.03; Figure 4D).

**Figure 5:**
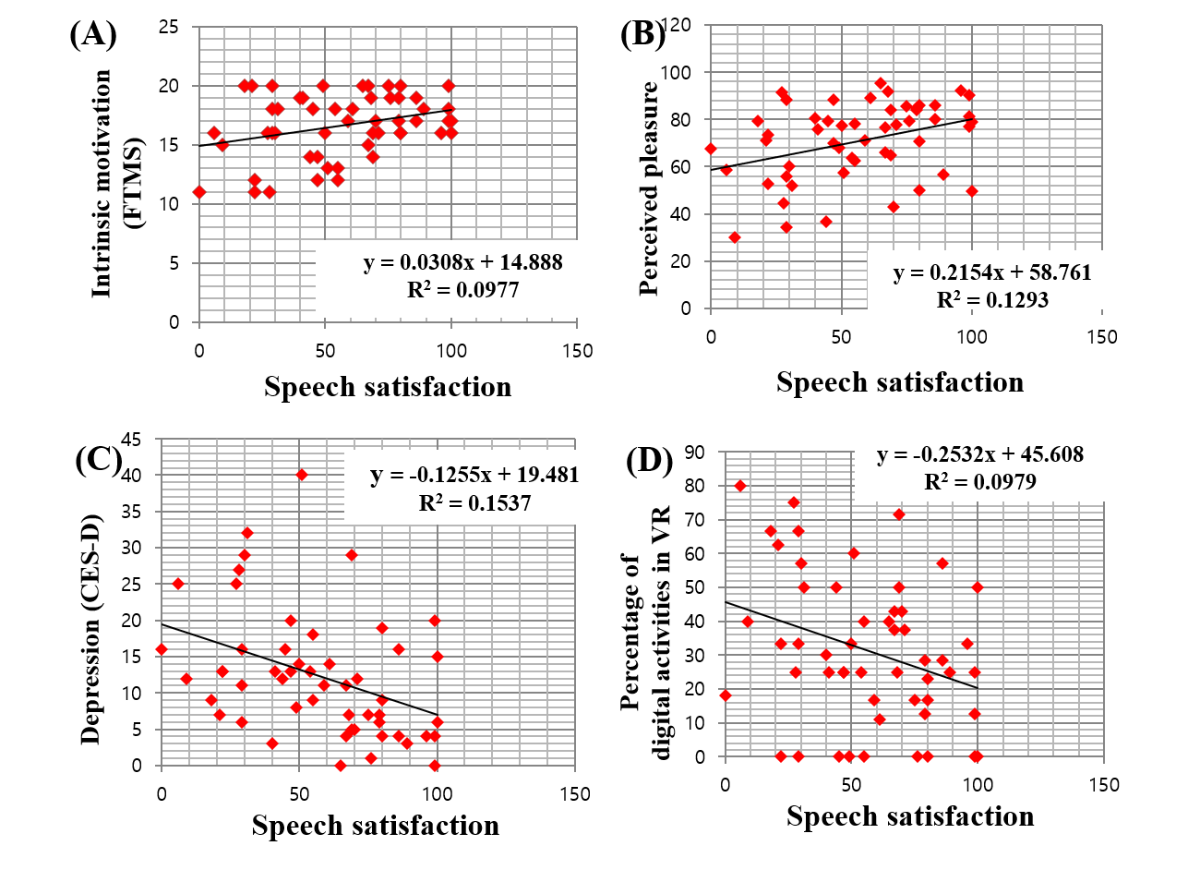
Correlation between speech satisfaction and (A) Free Time Motivation Scale (FTMS)-intrinsic motivation, (B) perceived pleasure, (C) depression, and (D) percentage of digital activities in virtual reality (VR). CES-D: Center for Epidemiological Studies Depression Scale.

The average speech satisfaction score in VR was positively correlated with motivation (r_52_=.312, *P*=.02) and perceived pleasure of the activity (r_52_=.31, *P*=.03; Figures 5A and 5B). Speech satisfaction score was negatively associated with depression (r_52_=−.392, *P*=.004) and frequency of digital activity in VR content (r_48_=.290, *P*=.04; Figures 5C and 5D). Independent *t* tests reported no significant differences in PQ, SSQ, and CSQ scores between the 2 groups. This shows that both IGD and control groups had similar experience in terms of immersion, simulator sickness, and program usability. 

## Discussion

Our VR program aimed to help IGD patients to identify their game-related behavior patterns by simulating the daily situations where they can select leisure activities and manage risky situations that lead to gaming. We also found significant relationships between the behaviors of youth with IGD in VR and in daily life by presenting the significant relationships among leisure activity selection patterns, built-in response data obtained from the VR program, and IGD symptom severity, psychiatric comorbidity, and motivation of participants reported through relevant questionnaire data. With varied VR content, users were expected to autonomously engage in self-help training with enhanced accessibility and without face-to-face supervision from therapists.

Based on CBT and MET principles, our VR program was constructed in a way that participants could practice managing malcognition about internet gaming and foster motivation and self-determination skills in an immersive virtual environment. Users were encouraged to think reflectively about their previous gaming behaviors and irrational game-related cognition. For example, one of the representative maladaptive patterns for cognition of patients with IGD who had “excessive preoccupation with internet gaming” was revealed through their overuse of digital-related activities, even in VR. Also, the real-time visual feedback report of selected activities using pie charts (Figure 1C) was intended to help patients to recognize their maladaptive leisure time behavior patterns and can potentially be useful to establish objective behavioral evidence to assess their IGD symptoms.

Moreover, the users were encouraged to increase their motivation to change by being given the opportunities for their own choice and self-direction while choosing free-time activities in our VR program. For example, the users were allowed to add their own activities in the Add Activity menu. Also, the self-rating component of perceived viability and perceived satisfaction of selected activities made it possible for the users to evaluate their motivation and acknowledge their own feelings related to this motivation. In addition, through self-speech in our VR program, users were given chances to voluntarily develop reflective thought-processing skills by considering the pros and cons of internet gaming.

The data from our VR program can provide information about daily activity patterns of youths with IGD, as well as the relationship between user VR activities and IGD symptoms. This suggests that IGD symptoms can influence the users’ selection patterns for leisure activities and ability to discover alternative activities to gaming. The finding that the IGD group had fewer leisure activities and preferred game or digital activities to other types of activities, even in VR, may reflect the lack of interest or pool of leisure activities in youth with IGD beyond games [15,16]. This was consistent with previous studies reporting cognitive bias (eg, attention and approach) to addiction-related stimuli in addiction-prone individuals [52]. Similarly, patients with IGD are known to have a general preoccupation with internet gaming and overvaluation of game-related activities and reward [15,16,53]. Our result was consistent with motivation-focused models postulating that addiction may be a disorder of misdirected motivation, in which greater priority is given to addiction-related stimuli [15,16,18].

Additionally, there was a positive correlation between the viability of alternative leisure activities participants selected and the amount of perceived satisfaction of the activities or user’s motivation. This suggests that it is important to train youths with IGD to recognize and engage in various types of alternative activities, rather than indulging only in internet gaming, during their leisure time. Through our VR program, individuals with IGD may broaden their range of leisure activities by getting more exposure to new alternatives to replace internet gaming. This finding also shows the potential of our VR-based training to predict and prevent IGD-related symptoms, by providing the opportunity for people to observe their behavior patterns in VR and practice selecting alternative activities of their own choice.

For IGD treatment, VR therapy is efficacious in decreasing craving and severity of internet addiction [54,55]. However, unlike VR therapy for substance addictions or pathological gambling, few studies have formally examined the efficacy of VR therapy for IGD, and evidence-based treatment components have not been well-established [13,56]. To base our program on clinically proven evidence, our program tried to incorporate the treatment component of traditional CBT and MET models of addiction [14,57]. Furthermore, adopting the motivational aspect of addiction, our results, such as the positive correlation between speech satisfaction or accordance and users’ motivation, negative correlation between speech satisfaction and the percentage of digital activities in the VR content, the IAT and IAT-gaming scores, and depressive symptoms indicating the behavior pattern, speech satisfaction, and speech accordance measured in our VR program, may be useful to assess motivation, IGD symptom severity. and affective aspects of youth [58].

Regarding the results of the PQ, SSQ, and CSQ, the IGD and control groups had similar levels of satisfaction in terms of immersion, simulator sickness, and program usability.

Although our findings showed that our VR may predict IGD-related symptoms through VR-based user activities, this study had several limitations to consider. The small sample size, inclusion of only male participants, and relatively mild level of IGD severity among our participants limit the generalizability of our findings to female populations and those with severe symptoms. Also, participants of a diverse age range were recruited for this study. Children, adolescents, and young adults may have their own unique characteristics in gaming motivation and various leisure activities. Therefore, a detailed subgroup analysis regarding the difference between age groups is needed in future studies. Moreover, 3 patients with comorbid psychiatric disorders were included, for which the presence of a comorbid condition may be a confounding factor. However, the main findings of our study generally remained the same as those when including participants with comorbidities. Additionally, symptoms of IGD or other psychopathologies were measured by self-report instruments, and objective behavioral observation of users or reports from caregivers are required. Also, the FTMS-A, originally developed for the adolescent population, was used for adult participants for the ease of comparison between adolescent and young adult groups; however, the use of a motivation scale for the adult population is still needed. Moreover, the measured variables of self-speech were limited to speech time or users’ subjective self-rated variables. For deeper qualitative analysis, meaning-based speech content analysis is required. Furthermore, with the current technical limitations, previously reported simulator sickness [50,59-61] made it difficult for people to experience VR content that exploit user motion or the implementation of a moving environment in VR. In addition, the avatar cannot give any verbal or nonverbal feedback to the users or afford any other form of interactivity; thus, our program was limited in terms of responsiveness and realism in the virtual interaction landscape. With future technical advancement in motion capture, face capture, speech recognition, and acoustic, meaning-based speech analysis, inputs from VR systems will be fed into an artificial intelligence model that can determine the user’s goals and provide appropriate outputs that can be translated into speech and gestures of avatars.

Also, some discrepancy may exist between leisure activities chosen in the VR and those in real life, as shown by the low speech accordance rate in IGD participants. Although our experiment was conducted in a controlled hospital setting with assistance from experimenters, further research is needed in the home environment using mobile or wearable devices to investigate the potential of our program as a standalone self-help therapy tool. Additionally, the role of gaming motivation as a mediating factor in the development of IGD was not fully explored in this study. It would be informative to investigate whether different motivations for playing internet games mediate or moderate the relationship between anxiety or depressive symptoms and IGD severity. Lastly, our preliminary study design did not allow for validation of our program to reduce IGD symptoms or related outcomes, such as the time spent on gaming or depressive symptoms, and various reliability measures, such as test-retest reliability, were also not measured. In future studies, different types of validity testing should be conducted with larger sample sizes to validate the clinical usefulness of our program.

Our results showed significant correlations between behaviors of adolescent and young adults with IGD in VR and in daily life, in terms of the selection and verbalization of their leisure activities and related cognition. If a database is accumulated for more precise speech content analysis with a larger sample size, this system can potentially serve as a useful tool for researchers to understand IGD more deeply and also have a considerable impact on designing interaction and interface in VR content for patients with IGD.

## Appendix

Multimedia Appendix 1 Virtual reality system and experimental environment.

Multimedia Appendix 2 Leisure activities in each room in Contents 1 and 2.

Multimedia Appendix 3 Screenshots for Content 2: (A) friend avatar's opinion; (B) self-speech; (C) self-report on accordance between speech and their opinion.

Multimedia Appendix 4 Script of Content 3a based on 3 gaming motivations.

## Abbreviations

ADHD: Attention Deficit Hyperactivity Disorder
BAI: Beck Anxiety Inventory
CASS-S: Conners-Wells’ Adolescent Self-Report Scale
CBT: cognitive behavioral therapy
CES-D: Center for Epidemiological Studies Depression Scale
CSQ: Client Satisfaction Questionnaire
DSM-5: Diagnostic and Statistical Manual of Mental Disorders, 5th edition
FTMS-A: Free Time Motivation Scale for Adolescents
IAT: Internet Addiction Test
IGD: internet gaming disorder
MET: motivational enhancement therapy
MINI-KID: Mini-International Neuropsychiatric Interview for Children and Adolescents
PQ: presence questionnaire
SSQ: Simulator Sickness Questionnaire
VAS: visual analog scale
VR: virtual reality
