# Supplementary figures and images for: Choice of Leisure Activities by Adolescents and Adults With Internet Gaming Disorder: Development and Feasibility Study of a Virtual Reality Program
Source: JMIR Serious Games. 2020 Dec 11;8(4):e18473. doi: 10.2196/18473 (PMC7762687; doi:10.2196/18473)

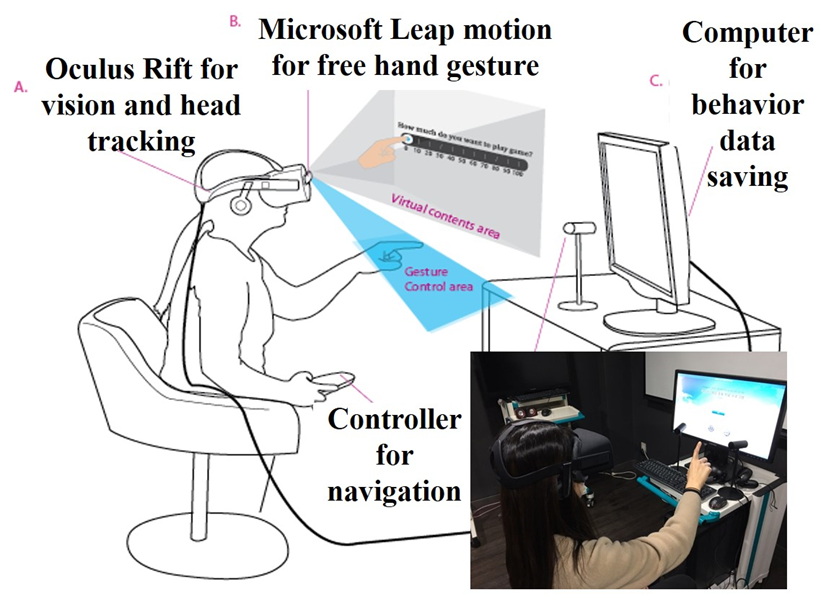

Supplement: Multimedia Appendix 1 [file games_v8i4e18473_app1.png]

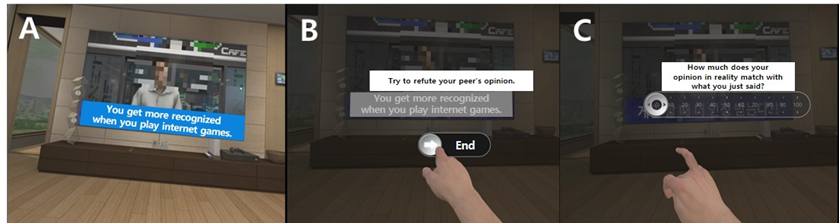

Supplement: Multimedia Appendix 3 [file games_v8i4e18473_app3.png]
