# Supplementary material for: Choice of Leisure Activities by Adolescents and Adults With Internet Gaming Disorder: Development and Feasibility Study of a Virtual Reality Program
Source: JMIR Serious Games. 2020 Dec 11;8(4):e18473. doi: 10.2196/18473 (PMC7762687; doi:10.2196/18473)
Supplement: Multimedia Appendix 2 [file games_v8i4e18473_app2.docx]

| Room | Activities (Code) |
| --- | --- |
| Living room | |
|  | TV (A—Drama [A01], Entertainment/Music [A02], Documentary [A03], News [A04], Educational [A05], Movie [A06], Animation [A07], Sports [A08], Online game [A09], Other [A10]), Pet (B), Board game (C), Go chess (D), Plamodel (E), Console game (F), Exercise (G) |
| Kitchen |  |
|  | Cooking Table (H), Kitchen Table (H2) |
| Small room | |
|  | Bed (I) |
| My room | |
|  | Bed (J), Newspaper (K), Magazine (L), Comic book (M), Closet (N), Mobile (O—Game [O01], Web surfing [O02], Internet community [O03], Web VOD [O04], TV [O05], Movie [O06], Music [O07], Web cartoon [O08], Radio [O09], SNS [O10], Video chat [O11], Other [O12]), Computer (P—Game [P01], News [P02], Internet community [P03], Web VOD [P04], TV [P05], Movie [P06], Music [P07], Web cartoon [P08], Radio [P09], SNS [P10], Other [P11]), Musical instrument (Q), Yoga mat (R), Art supply (S), Vacuum cleaner (T), Textbook (U) |
| Bathroom | |
|  | Bathtub (V), Cosmetic (W) |
| Veranda | |
|  | Watering plants (X) |
