# Supplementary material for: Choice of Leisure Activities by Adolescents and Adults With Internet Gaming Disorder: Development and Feasibility Study of a Virtual Reality Program
Source: JMIR Serious Games. 2020 Dec 11;8(4):e18473. doi: 10.2196/18473 (PMC7762687; doi:10.2196/18473)
Supplement: Multimedia Appendix 4 [file games_v8i4e18473_app4.docx]

| Gaming motivation | Avatar’s claims | Script in Content 3^a^ |
| --- | --- | --- |
| Socialization^b^ | I have close relationships with my friends. | I have friends whom I meet in games to play together. We are always there to help or save each other. I only have a strong attachment toward friends in games as opposed to friends in the real world. |
|  | Friends online are more friendly. | I feel more connected to friends I meet in online games. I can only depend on my online friends to talk about annoying problems and worries. |
| Dissociation^c^ | I can forget a bad feeling. | When I feel upset, get scolded, or don't want to study for mid-terms, online gaming is the only way to forget about negative feelings. |
|  | I can forget things I should do but don’t want to. | When I don’t want to do something that I have to do, concentrating on an online game is the only way to forget about the annoying reality. |
| Achievement^d^ | I can feel a sense of achievement. | If I win an Internet game, my winning rate or level within the game rises. Although my level in the game is very high right now, achieving an even higher level is the only way I can feel good about myself. |
|  | When I play games, I feel more acknowledged. | I played a leading role in winning the team championship. A member in the same team then gave me recognition, which makes me feel prouder than anything else. |

^a^ The structure of this script was adopted from the concept of gaming motivation from Ryan and Deci (2000a, 2000b).

^b^Socialization (ie, to make friends and provide mutual support while playing the game).

^c^achievement (ie, seeking to achieve one’s goals during the game).

^d^dissociation (ie, to avoid/escape reality while playing the game).
